# Promoter hypermethylation of *RARB* and *GSTP1* genes in plasma cell‐free DNA as breast cancer biomarkers in Peruvian women

**DOI:** 10.1002/mgg3.2260

**Published:** 2023-08-07

**Authors:** Pierina Danos, Stefano Giannoni‐Luza, Alexis Germán Murillo Carrasco, Oscar Acosta, Maria Luisa Guevara‐Fujita, José Manuel Cotrina Concha, Henry Guerra Miller, Joseph Pinto Oblitas, Alfredo Aguilar Cartagena, Jhajaira M. Araujo, Ricardo Fujita, José Luis Buleje Sono

**Affiliations:** ^1^ Centro de Genética y Biología Molecular Universidad de San Martín de Porres Lima Peru; ^2^ Facultad de Medicina Humana Universidad de San Martín de Porres Chiclayo Peru; ^3^ Facultad de Farmacia y Bioquímica Universidad Nacional Mayor de San Marcos Lima Peru; ^4^ Instituto Nacional de Enfermedades Neoplásicas (INEN) Lima Peru; ^5^ Oncosalud‐Auna Lima Peru

**Keywords:** breast cancer, cfDNA, *GSTP1*, liquid biopsy, *Methylight*, PMR, *RARB*

## Abstract

**Background:**

Promoter hypermethylation is one of the enabling mechanisms of hallmarks of cancer. Tumor suppressor genes like *RARB* and *GSTP1* have been reported as hypermethylated in breast cancer tumors compared with normal tissues in several populations. This case–control study aimed to determine the association between the promoter methylation ratio (PMR) of *RARB* and *GSTP1* genes (separately and as a group) with breast cancer and its clinical‐pathological variables in Peruvian patients, using a liquid biopsy approach.

**Methods:**

A total of 58 breast cancer patients and 58 healthy controls, matched by age, participated in the study. We exacted cell‐free DNA (cfDNA) from blood plasma and converted it by bisulfite salts. *Methylight* PCR was performed to obtain the PMR value of the studied genes. We determined the association between PMR and breast cancer, in addition to other clinicopathological variables. The sensitivity and specificity of the PMR of these genes were obtained.

**Results:**

A significant association was not found between breast cancer and the *RARB* PMR (OR = 1.90; 95% CI [0.62–6.18]; *p* = 0.210) or the *GSTP1* PMR (OR = 6.57; 95% CI [0.75–307.66]; *p* = 0.114). The combination of the *RARB + GSTP1* PMR was associated with breast cancer (OR = 2.81; 95% CI [1.02–8.22]; *p* = 0.026), controls under 50 years old (*p* = 0.048), patients older than 50 (*p* = 0.007), and postmenopausal (*p* = 0.034). The PMR of both genes showed a specificity of 86.21% and a sensitivity of 31.03%.

**Conclusion:**

Promoter hypermethylation of *RARB + GSTP1* genes is associated with breast cancer, older age, and postmenopausal Peruvian patients. The methylated promoter of the *RARB + GSTP1* genes needs further validation to be used as a biomarker for liquid biopsy and as a recommendation criterion for additional tests in asymptomatic women younger than 50 years.

## Graphical abstract

The promoter hypermethylation of RARβ2+GSTP1 is associated with breast cancer, older age, and postmenopausal patients. The PMR of both genes showed a specificity of 86.21% and a sensitivity of 31.03%. Our study accounts for the first evidence of the association of promoter hypermethylation of RARB2 and GSTP1 genes in liquid biopsies (circulating cfDNA) and breast cancer in Peruvian women.

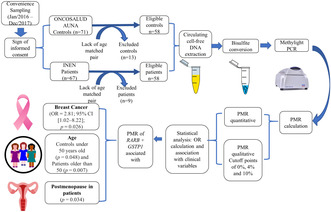

## 1 INTRODUCTION

Promoter hypermethylation of tumor suppressor genes occurs generally during the asymptomatic stage and is considered a promising early biomarker of breast cancer (Esteller, 2008; Johnson et al., 2014). Assessment of DNA methylation involves the distribution of methylated and unmethylated CpG islands in the gene promoter and other regions of the genome, such as heterochromatin and centromeres (Esteller, 2008). Epigenetic changes are very stable, and some gene promoters have been reported methylated in most breast cancer tumors (Fujita et al., 2012; Song et al., 2017).

Although aberrant DNA methylation is an early event in the development of breast cancer, this association is not well characterized (Johnson et al., 2014). The extent to which changes in methylation are characteristic of healthy tissue and what changes lead to the development of cancer remain unknown. However, the hypermethylation of different gene promoters is a common feature of this scenario (Song et al., 2017). Methylated DNA patterns have the potential to identify tumor cells, discerning them from healthy tissue. Their use is currently considered a diagnostic biomarker (Fujita et al., 2012; Szyf, 2012).

Recently, free circulating DNA (cfDNA) from blood has proven to be a useful source of these biomarkers by containing tumor‐derived DNA. In contrast with tissue biopsies, liquid biopsies are and do not involve complex and time‐delaying procedures. In addition, the cfDNA would exhibit the same tumoral mutations and epigenetic changes, with minimal interference from leukocyte DNA (Warton & Samimi, 2015).

In particular, promoter methylation of *RARB* and *GSTP1*, both tumor suppressor genes, counted with meta‐analysis evidence of association with breast cancer in Caucasian, African, and Asian populations (Fang, Jian, et al., 2015; Fang, Wei, et al., 2015). Notably, methylation assessment in cancer patients from Hispanic or Native American populations is underrepresented. In this context, the Peruvian population from Lima offers an average genetic background of 70% Native American component (Sandoval et al., 2013).

Following, *RARB (OMIM:180220)* induces cellular apoptosis and has anti‐proliferative functions in the presence of retinoic acid. Methylation of *RARB* promoter would condition a favorable environment for cancer progression. Hypermethylation of *RARB* promoter has been found to be associated with breast cancer using total DNA from peripheral blood samples and tissue (Fang, Jian, et al., 2015). Similarly, *GSTP1 (OMIM:134660)* expresses an enzyme involved in cell detoxification that can interact with regulatory kinases and direct pathways related to cell differentiation, proliferation, and death. Promoter hypermethylation of GSTP1 has also been significantly associated with breast cancer in samples coming from the blood and tissue of patients (Fang, Wei, et al., 2015).

On the other hand, tumor gene expression profiles have generated intrinsic subtypes of breast cancer that provide a molecular identity to the tumor and are indicators of recurrence, survival, and response to treatment (Szyf, 2012). However, genetic markers tend to be heterogeneous and dispersed throughout the genome. This diversity makes the development of a single genetic screening test for breast cancer difficult (Warton et al., 2014).

Moreover, promoter regions of candidate genes like *GSTP1* and *RARB* are hypermethylated in most patients with breast cancer, regardless of the cancer stage (Shukla et al., 2006; Yamamoto et al., 2012). Nevertheless, the clinical usage of methylated biomarkers in breast cancer diagnosis has not been implemented to date due to several factors, including the methodological challenges of working with bisulfite‐converted DNA (de Ruijter et al., 2020).

In 2020, breast cancer had the highest incidence in Peruvian women (18.5%) at an age‐standardized incidence rate of 35.9 years. This disease was also the most prevalent during the last 5 years (Sung et al., 2021). When detected prematurely, the survival rate of breast cancer in the early stages is 98%, while in metastatic stages, the rate drops to 27% (Radpour et al., 2011). Accordingly, the strategies used by the Peruvian Ministry of Health (Minsa) to prevent and treat breast cancer prioritize early detection (Zelle et al., 2013).

Therefore, there is a need to establish a breast cancer detection panel that includes epigenetic biomarkers for adequate screening in Peru. Due to the lack of epigenetic biomarkers tested in our population, we designed a case–control study to assess the methylation frequencies of two well‐known tumor suppressor genes. The promoter methylation ratio (PMR) found in patients and healthy women, paired by age, was associated with the diagnosis of cancer and clinicopathological characteristics (age, hormonal status, tumor stage, immunohistochemistry, molecular subtypes). The sensitivity and specificity of these markers were also assessed.

## 2 METHODS

### 2.1 Ethical compliance

The informed consent and study protocol were approved by the Protocols Review Committee from INEN and Universidad de San Martín de Porres IRB (IRB00003251‐FWA0015320), issued on June 22, 2015, with legal number 826–2015–CIEI–USMP–CCM.

### 2.2 Sample size calculation

Power calculation for matched case–control studies were done using Epidat 3.1 (Xunta de Galicia Direccion Xeral de Saúde Publica Consellería de Sanidade, 2004) and the following parameters: for *RARB*, the probability of exposure to breast cancer for women was 37.32% and the expected odds ratio (OR) of 7.27 (Fang, Jian, et al., 2015). For *GSTP1*, the probability of exposure to breast cancer was 33.45% and an expected OR of 7.85 (Fang, Wei, et al., 2015). With a given alpha of 5%, the required number of pairs for a matched sample size was 33 for *RARB* and 35 for *GSTP1* to reach a statistical power of 80%. Statistical parameters for sample size and power calculation can be found in Table S1.

### 2.3 Patients and sample collection

Samples and clinical history were obtained in collaboration with *Instituto Nacional de Enfermedades Neoplásicas* (INEN) and the *Oncoslud‐AUNA* network. Breast cancer phenotype classification was based on the 2011 St. Gallen International Expert Consensus (Kondov et al., 2018). Healthy control samples came from the *Oncosalud‐AUNA* Breast Cancer Prevention Program, previously diagnosed without any malign tumor in the breast tissue.

**FIGURE 1:**
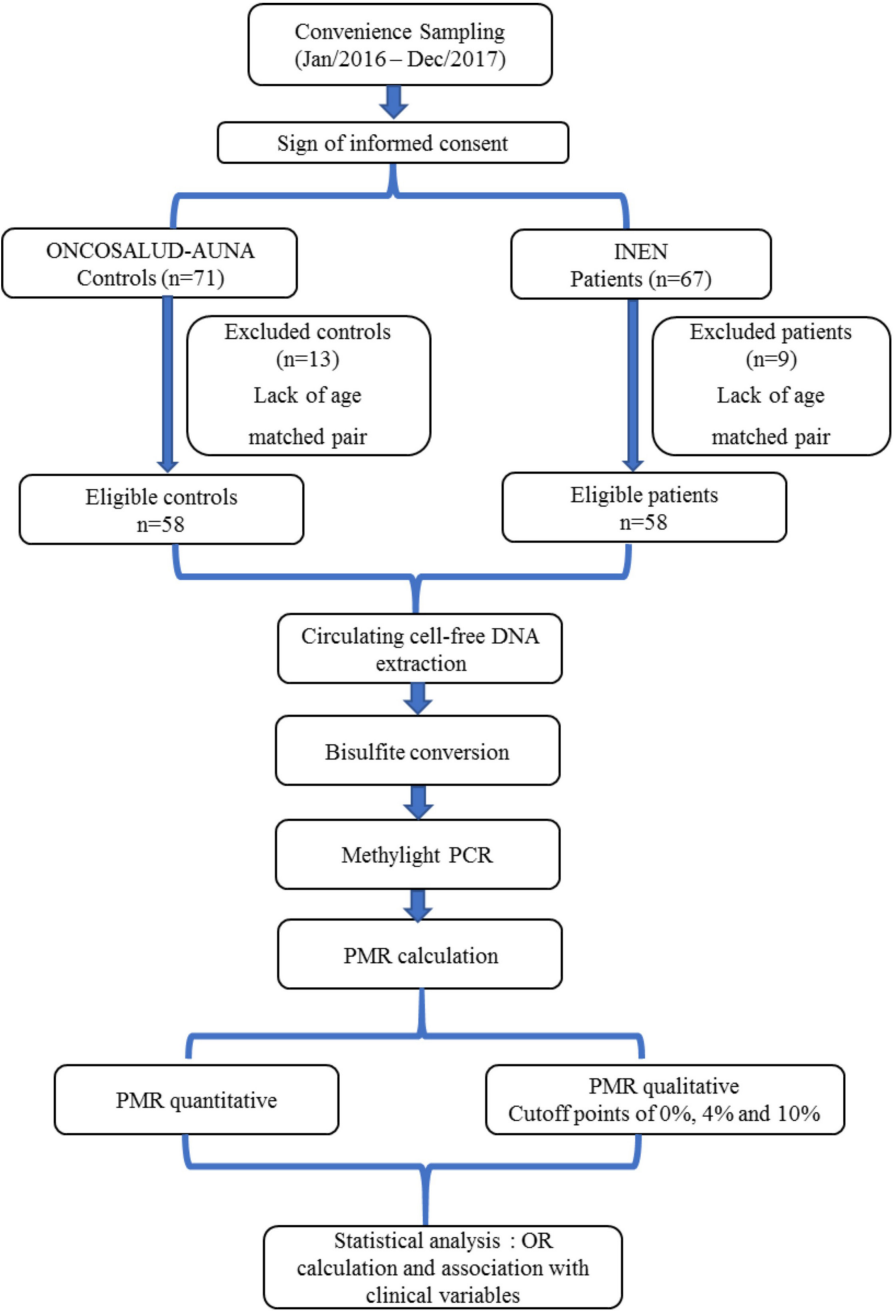
Flowchart of the study.

A convenience sampling was performed between January 2016 and December 2017, enrolling 71 healthy controls and 67 patients with breast cancer. Of these, 58 patients and 58 paired controls (*n* = 116) fulfilled the inclusion criteria, which were being a woman diagnosed with breast cancer at any stage and having an age‐matched control with a maximum of 2 years difference. Samples were therefore anonymized and sent to a core Laboratory (*Centro de Investigación en Genética y Biología Molecular*, *CIGBM*) at Universidad de San Martin de Porres for further analysis. A flowchart of the methodology is depicted in Figure 1.

### 2.4 Circulating cell‐free DNA extraction

Liquid biopsy samples consisted of 3 mL of total blood in a BD Vacutainer® tube with EDTA K2. Plasma was separated by refrigerated centrifugation and stored at −80°C until analysis. cfDNA was extracted using the NucleoSpin Plasma XS Kit (Macherey‐Nagel) from 750 μL of plasma according to the manufacturer's specifications, eluted in 50 μL of 10:1 TE buffer, and quantified by NanoDrop™ Lite Spectrophotometer (Thermo Scientific™) (Thermo Fisher Scientific, 2011). Qiagen Epitect Bisulfite kit was used according to the manufacturer's protocol for severely fragmented samples (Qiagen, 2014). Bisulfite‐converted DNA was eluted in 40 μL of 10:1 TE buffer and was then separated into two aliquots of 20 μL each, to avoid further degradation by repetitive thawing. Aliquots were stored at −80°C until performing the *Methylight* assay.

### 2.5 Primers and probes

For the endogenous reference gene *COL2A1* and the study genes, *GSTP1* and *RARB*, we used the primers and probe for methylated promoter described in Fujita et al. (2014). The gene identifiers and oligonucleotides considered for the assay are described in Table S2. All probes were designed with the FAM dye. Oligonucleotides were diluted to 100 μM Stock and working aliquots were diluted to 10 μM.

### 2.6 *Methylight* PCR

We used the methodology described in Dallol et al. (2011) to design a *Methylight* PCR, considering batches of eight samples. Briefly, we designed a standard curve using *COL2A1* as the endogenous reference gene. Control DNA 100% methylated from the Epitect control kit (Qiagen) was used to prepare a standard curve with four points in triplicate concentrations as follows: A (2 ng/μL), B (1 ng/μL), C (0.5 ng/μL), and D (0.25 ng/μL). We used fully methylated DNA at a concentration of 0.25 ng/μL for positive control of *GSTP1* and *RARB* (called “B”) and *COL2A1* (called “D”). The 10X Primer‐Probe Mix (10X PPM) consisted of 4 μM forward primer, 4 μM reverse primer, and 2 μM probe for each gene. The following reaction controls were also included: “E” (100% unmethylated control DNA converted by bisulfite at 0.5 ng/μL) that amplifies only for *COL2A1* and “F” (100% unmethylated control DNA not converted by bisulfite at 0.5 ng/μL) as negative control. The final reaction volume of 20 μL and program conditions for Qiagen Rotor‐Gene Q equipment are described in Table S3. Due to the fragmented and difficult nature of the sample, amplification curves with a Ct after 45 cycles were considered artifacts and negative amplification. Gene amplification was validated on each assay with control B in duplicate. We used Rotor‐Gene Q Series Software to obtain the concentration of the *GSTP1* and *RARB* genes on each run.

### 2.7 Statistical analysis

Data were exported to a Microsoft Excel 2016 spreadsheet, where the PMR for each gene was calculated. We calculated the PMR using the formula (adapted from Dallol et al., 2011, which gives the ratio of promoter methylation in percentage units (quantitative data).

$$\begin{array}{c} \text{Sample}\;\mathrm{PMR}=\frac{\frac{A}{B}}{\frac{C}{D_{\;}}}\\ =\frac{\frac{\text{Test gene}\;\left(\text{GSTP}1\;\text{or}\;\text{RARB}\right)\;\text{levels in the patient sample}}{\text{Test gene}\;\left(\text{GSTP}1\;\text{or}\;\text{RARB}\right)\;\text{levels in the}\;100\%\text{methylated control}}}{\frac{\;\mathrm{COL}2A1\;\text{gene levels in the patient sample}}{\;\mathrm{COL}2A1\;\text{gene levels in the}\;100\%\text{methylated control}}}*100\% \end{array}$$

The PMR value obtained for each gene was analyzed in its quantitative and qualitative form. Additionally, qualitative PMR was analyzed considering cut‐off values of 4% (Ogino et al., 2006) and 10% (Dallol et al., 2011) of promoter methylation. As suggested by previous authors, PMR distribution tends to be bimodal and the use of arbitrary cut‐off points needs more examination (Dallol et al., 2011).

PMR values were organized in a database with the clinical information of each participant and the association between variables was analyzed using the STATA v14 software (StataCorp., 2015).

Since quantitative PMR has non‐normal distribution, we tested the association of breast cancer and PMR of *RARB2* and *GSTP1* genes using a two‐sample Wilcoxon rank‐sum (Mann–Whitney) test. The odds ratio for breast cancer and the qualitative PMR values were determined using chi‐squared or Fisher exact test. Also, OR was obtained using logistic regression of fixed effects for cases and controls matched by age.

Quantitative PMR and its association with clinicopathological variables were obtained using Mann–Whitney's *U* and Kruskal–Wallis test. The association of qualitative PMR values and clinicopathological variables of breast cancer patients was obtained with the chi‐squared test and Fisher's exact test. Lastly, sensitivity, specificity, and predictive values for the PMR of the RARB2 and GSTP1 were calculated using Epidat 4.2 software (Xunta de Galicia Direccion Xeral de Saúde Publica Consellería de Sanidade, 2004) and breast cancer clinical diagnosis as the gold standard.

## 3 RESULTS

### 3.1 Patients characteristics

The demographic characteristics of participants are listed in Table 1. Of the group of patients, 69% were postmenopausal; the most frequent stage of cancer was stage III with 48.3%, followed by stage II with 23.2%, stage I with 16%, and stage IV with 12.5%. Regarding immunohistochemical markers, 64.3% of the patients expressed estrogen receptor (ER), 51.8% of the patients were positive for the progesterone receptor (PR) and only 25.4% were HER2 positive. Luminal A was the most frequent molecular subtype (58.93%), followed by the HER2 enriched (19.64%), basal‐like subtype (14.29%), and Luminal B (7.14%).

**TABLE 1:** General and clinical characteristics of breast cancer patients and controls.

	Controls *n* (%)	Patients *n* (%)
*n* = 116	58 (50)	58 (50)
**Age**	***n* = 58**	***n* = 58**
Mean	52.93	52.93
Median	52.00	52.00
95% CI	[49–56.90]	[49.05–57]
Range	[20–85]	[21–80]
**Initial DNA mass (ng)**	***n* = 39**	***n* = 36**
Mean	427.90	455.45
Median	399.90	424.00
95% CI	[399.50–402.90]	[298.90–515.70]
Range	[156.25–1292]	[99.84–1268]
**COL2A1 (ng/reax)**	***n* = 58**	***n* = 58**
Mean	0.45	0.47
Median	0.33	0.39
95% CI	[0.20–0.40]	[0.24–0.50]
Range	[0.01–1.65]	[0.05–4.46]
**Hormonal status**		***n* = 58**
Premenopausal		24 (41.38)
Postmenopausal		40 (68.97)
**Cancer stage**		***n* = 56**
I		9 (16.07)
II		13 (23.21)
III		27 (48.21)
IV		7 (12.50)
**Initial and final stage**		***n* = 56**
Initial stage (I y II)		22 (39.29)
Final stage (III y IV)		34 (60.71)
**Estrogen receptor (ER)**		***n* = 56**
Negative		20 (35.71)
Positive		36 (64.29)
**Progesterone receptor (PR)**		***n* = 56**
Negative		27 (48.21)
Positive		29 (51.79)
**HER2**		***n* = 55**
Negative		41 (74.55)
Positive		14 (25.45)
**Molecular subtypes**		***n* = 56**
Luminal A		33 (58.93)
Luminal B		4 (7.14)
Basal like		8 (14.29)
HER2 enriched		11 (19.64)
**Hormone receptor positive**		***n* = 56**
Negative		19 (33.93)
Positive		37 (66.07)
**Triple negative**		***n* = 56**
Negative		48 (85.71)
Positive		8 (14.29)

The average values of cfDNA and *COL2A1* obtained are shown in Table S4. No significant difference was found in the initial DNA mass used for bisulfite conversion between the patient and control groups (*p* = 1.000). Following, the average *COL2A1* concentration (ng/reax) of patients and controls was similar after the *Methylight* PCR (*p* = 0.945).

### 3.2 PMR analysis

As shown in Table 2, amplification of *RARB* gene promoter was obtained in 12 (63.16%) patients and seven (36.84%) controls. The *GSTP1* gene promoter was found methylated in six (85.71%) patients and one (14.29%) control. As a panel, *RARB + GSTP1* methylation was found in 18 (69.23%) patients and in eight (30.77%) controls.

**TABLE 2:** Association of the PMR of *GSTP1* and *RARB* genes and the risk of breast cancer.

*N* = 116	Control	Patient	Test	*p*‐Value
Quantitative PMR (%)				
PMR *RARB*				
Mean	6.77	4.11	−1.1000	0.269**
Median [Range]	0 [0–163.31]	0 [0–73.94]		
PMR *GSTP1*				
Mean	0.42	4.64	−1.9400	0.052**
Median [Range]	0 [0–24.72]	0 [0–146.95]		
PMR RARB + GSTP1				
Mean	7.19	8.75	−2.065	0.039**
Median [Range]	0 [0–163.31]	0 [0–146.95]		

	Control (%)	Patient (%)	Total	OR	CI 95%	*p*‐Value	Adjusted OR	CI 95%	*p*‐Value
Qualitative PMR									
PMR RARB									
Methylated	7 (36.84)	12 (63.16)	19	1.90	[0.62–6.18]	0.210	2	[0.68–5.85]	0.192
Unmethylated	51 (52.58)	46 (47.42)	97						
PMR GSTP1									
Methylated	1 (14.29)	6(85.71)	7	6.57°	[0.75–307.66]	0.114	6	[0.72–49.83]	0.047
Unmethylated	57 (52.29)	52(47.71)	109						
PMR RARB + GSTP1									
Methylated	8 (30.77)	18(69.23)	26	2.81	[1.02–8.22]	**0.026**	2.7	[1.04–6.8]	**0.030**
Unmethylated	50 (55.56)	40(44.44)	90						
Qualitative PMR—Cut‐off 4%									
PMR RARB									
Methylated	7 (41.18)	10 (58.82)	17	1.52	[0.48–5.08]	0.431	1.6	[0.52–4.89]	0.403
Unmethylated	51 (51.52)	48 (48.48)	99						
PMR GSTP1									
Methylated	1 (14.29)	6 (85.71)	7	6.57°	[0.75–307.66]	0.114	6	[0.72–49.83]	0.047
Unmethylated	57 (52.29)	52 (47.71)	109						
PMR RARB + GSTP1									
Methylated	8 (33.33)	16 (66.67)	24	2.38	[0.85–7.05]	0.067	2.33	[0.89–6.07]	0.070
Unmethylated	50 (54.35)	42 (45.65)	92						
Qualitative PMR—Cut‐off 10%									
PMR RARB									
Methylated	5 (41.67)	7 (58.33)	12	1.45	[0.37–6.19]	0.542	1.4	[0.44–4.41]	0.563
Unmethylated	53 (50.96)	51 (49.04)	104						
PMR GSTP1									
Methylated	1 (14.29)	6 (85.71)	7	6.57°	[0.75–307.66]	0.114	6	[0.72–49.836]	0.047
Unmethylated	57 (52.29)	52 (47.71)	109						
PMR RARB + GSTP1									
Methylated	6 (31.58)	13 (68.42)	19	2.50	[0.80–8.66]	0.079	2.16	[0.82–5.70]	0.104
Unmethylated	52 (53.61)	45 (46.39)	97						

*Note*: Association of breast cancer and methylation of RARB and GSTP1 genes was determined. **p‐value obtained using Two‐sample Wilcoxon rank‐sum (Mann–Whitney) test. OR was determined using chi‐squared or exact test. Adjusted OR was obtained using logistic regression of fixed effects for cases and controls matched by age. Significant *p*‐values are shown in bold.

Abbreviations: PMR, promoter methylation ratio. Percentage units; CI, confidence interval; OR, odds ratio.

Following, no association was found between quantitative PMR of *RARB* or *GSTP1* and breast cancer (*p*‐value>0.05). When analyzed together, the PMR values of both genes were significantly associated with breast cancer (*p* = 0.039).

Similarly, qualitative PMR of *RARB* and breast cancer showed no association (OR = 1.90; 95% CI [0.62–6.18]; *p* = 0.210), which was confirmed by the adjusted OR (*p* > 0.05).

The qualitative PMR of *GSTP1* showed no association with breast cancer (OR = 6.57 95% CI [0.75–307.66]; *p* = 0.114). The adjusted OR for GSTP1 (OR = 6.0, 95% CI [0.72–49.83]) showed the CI included unity and the *p* = 0.047 at the limit of significance (Table 2), we may attribute this effect to variability in data that could affect its distribution. Thus, this *p*‐value was considered not significant.

Furthermore, the association of qualitative PMR of both genes (*RARB + GSTP1*) with breast cancer was estimated. Patients with breast cancer were almost three times more likely to have been exposed to *RARB* and *GSTP1* methylation than controls (OR = 2.81; 95% CI [1.02–8.22]; *p* = 0.026). However, this association was lost when using the PMR threshold of 4% and 10%.

Considering the intrinsic variables of controls and patients, no association was obtained between the age of the controls and the qualitative PMR of *RARB* (*p* = 0.102) or GSTP1 (*p* = 0.397). On the other hand, the qualitative PMR of both genes (*RARB + GSTP1*) was associated with age younger than 50 (*p* = 0.048) in healthy controls, as shown in Table 3. Further, a PMR of *RARB* above 10% was associated with age older than 50 in the patients. No other clinical‐pathological variable of the patients was associated with methylated *RARB* or *GSTP1*. Considering the qualitative PMR of both genes (*RARB + GSTP1*), the association was found with patients older than 50 years (*p* = 0.007) and postmenopausal hormonal status (*p* = 0.034) as shown in Table 3. Additionally, the quantitative PMR of (*RARB + GSTP1*) was also associated with age above 50 (*p* = 0.017) and postmenopausal hormonal status (*p* = 0.023).

**TABLE 3:** Association between clinicopathological variables of breast cancer patients and promoter methylation ratio (PMR) of the *RARB2 + GSTP1* panel.

	Qualitative PMR			
	*N* = 58	PMR (−), *n* (%)	PMR (+), *n* (%)	*p*‐Value
Controls				
Age				
<50 years	23 (39.66)	17 (34)	6 (75)	**0.048** *
≥50 years	35 (60.34)	33 (66)	2 (25)	
Patients				
Age				
<50 years	22 (100)	20 (90.91)	2 (9.09)	**0.007** *
≥50 years	36 (100)	20 (55.56)	16 (44.44)	
Hormonal status				
Premenopausal	18 (31.03)	16 (40)	2 (11.11)	**0.034** *
Postmenopausal	40 (68.97)	24 (60)	16 (88.89)	
Cancer Stage				
I	9 (16.07)	6 (15.38)	3 (17.65)	0.357*
II	13 (23.21)	9 (23.08)	4 (23.53)	
III	27 (48.21)	21 (53.85)	6 (35.29)	
IV	7 (12.50)	3 (7.69)	4 (23.53)	
Initial and final stage				
Initial stage (I y II)	22 (39.29)	15 (38.46)	7 (41.18)	0.848*
Final stage (III y IV)	34 (60.71)	24 (61.54)	10 (58.82)	
Estrogen receptor (ER)				
Negative	20 (35.71)	12 (30.77)	8 (47.06)	0.242
Positive	36 (64.29)	27 (69.23)	9 (52.94)	
Unknown				
Progesterone receptor (PR)				
Negative	27 (48.21)	17 (43.59)	10 (58.82)	0.294
Positive	29 (51.79)	22 (56.41)	7 (41.18)	
Unknown				
HER2				
Negative	41 (74.55)	28 (73.68)	13 (76.47)	0.826
Positive	14 (25.45)	10 (26.32)	4 (23.53)	
Unknown				
Molecular subtypes				
Luminal A	33 (58.93)	24 (61.54)	9 (52.94)	0.366*
Luminal B	4 (7.14)	4 (10.26)	0 (0)	
Basal like	8 (14.29)	4 (10.26)	4 (23.53)	
HER2 enriched	11 (19.64)	7 (17.95)	4 (23.53)	
Hormone receptor positive				
Negative	19 (33.93)	11 (28.21)	8 (47.06)	0.171
Positive	37 (66.07)	28 (71.79)	9 (52.94)	
Triple negative				
Negative	48 (85.71)	35 (89.74)	13 (76.47)	0.228*
Positive	8 (14.29)	4 (10.26)	4 (23.53)	

* The *p*‐value was obtained with the chi‐squared test and Fisher's exact. Significant *p*‐values are shown in bold.

### 3.3 Sensitivity and specificity

*Methylight* assay of liquid biopsies was compared to medical diagnosis (gold standard). The specificity and sensitivity of the studied genes are shown in Table 4. The highest sensitivity (31.03%) was obtained using both genes (*RARB + GSTP1*), with a specificity of 86.21%.

**TABLE 4:** Sensitivity, specificity, and predictive values for the promoter methylated ratio (PMR) of the *RARB, GSTP1* genes, and as panel (*RARB + GSTP1*).

	Patients (*n* = 58) (%)	Controls (*n* = 58)	Sensitivity (%)**	95% CI	Specificity (%)**	95% CI	PPV (%)	NPV (%)
PMR *RARB*								
Methylated	12 (63.16)	7 (36.84)	20.69	[9.4–31.98]	87.93	[78.69–97.18]	63.16	52.58
Unmethylated	46 (47.42)	51 (52.58)						
PMR *GSTP1*								
Methylated	6 (85.71)	1 (14.29)	10.34	[1.65–19.04]	98.28	[94.06–100]	85.71	52.29
Unmethylated	52 (47.71)	57 (52.29)						
PMR *RARB + GSTP1*								
Methylated	18 (69.23)	8 (30.77)	31.03	[18.27–43.8]	86.21	[76.47–95.94]	69.23	55.56
Unmethylated	40 (44.44)	50 (55.56)						

*Note*: Clinical breast cancer diagnosis was used as the gold standard for calculating sensitivity and specificity.

Abbreviations: NPV, negative predictive value; PPV, positive predictive value.

** Calculated with Epidat 3.1—Simple Diagnostic Test.

## 4 DISCUSSION

Cancer is a complex process that involves a multitude of molecular alterations that synergistically contributes to tumor onset and development. Among these, epigenetic changes of tumor‐associated genes have been observed to contribute as drivers of homeostasis disruption. Deregulation of CpG methylation patterns, just as the hypermethylation of tumor suppressor genes, has been recognized as a hallmark of cancer (Berdasco & Esteller, 2019). Among these, promoter methylation of *RARB* and *GSTP1* genes has shown the potential to be used as breast cancer biomarkers.

PMR of *RARB* and *GSTP1* genes were obtained from blood plasma cfDNA of 58 breast cancer patients and 58 controls from Oncosalud‐Auna and INEN. No significant differences were found in the age of patients and controls, considering the age‐matching inclusion criteria. Nonetheless, the hormonal status of the controls was not considered in the medical record at the time of enrollment, and this could not be retrieved.

In the present study, the lowest DNA mass value detected for *COL2A1* amplification was 10 pg/reaction, enough to detect a minimum of one to two genomic copies of methylated DNA per assay (Pedersen et al., 2014). PCR template amount highly influences results in a *Methylight* analysis. Therefore, bisulfite‐treated input DNA should not vary significantly within the same experiment or between different *Methylight* assays (Pharo et al., 2016). The absence of significant differences in the initial cfDNA mass amount (ng) that was used in the bisulfite conversion and the *COL2A1* concentration (ng/reax) between the patient and control groups in all the assays, indicated a good reproducibility of the performed *Methylight* assays.

Fragmentation and degradation of *cf*DNA may highly increase after bisulfite salt conversion, increasing the likelihood of destroying the binding site of the probe or primers. However, it is necessary to assess the integrity of the extracted *cf*DNA to support this claim. The presence of methylation heterogeneity should be considered in further study design since it could contribute to the variability of the results obtained from different studies (Loke & Lee, 2018; Shukla et al., 2006).

Reduced frequency of promoter methylation of *RARB2* and *GSTP1* in liquid biopsy samples compared with tumors has been reported in different studies (Fang, Wei, et al., 2015; Gurioli et al., 2018). This difference accounts for *cf*DNA, which has a greater degree of fragmentation than lymphocytic and tumor DNA, along with a lower concentration of the targeted sample (Esteller, 2008). This effect may explain the loss of association using cut‐off points of 4% and 10% PMR in our sample, due to a less available template of methylated DNA.

However, we obtained a frequency of 20.7% of methylated patients for *RARB* and 10.3% of methylated patients for *GSTP1*. Considering that the hypermethylation of these genes in serum and tumors is variable (Fujita et al., 2012; Shukla et al., 2006), these low frequencies would be explained due to the presence of false‐negative results because of the low amount of tumor templates in the cfDNA from plasma (Brooks et al., 2010). As a limitation of this study, tumoral biopsies of methylated patients were not available to confirm the methylation status found in the liquid biopsy samples. Further methylation analysis of breast tumor biopsy is needed to determine the methylation frequencies of these genes in the Peruvian population.

Meta‐analysis made by Fang et al. using total DNA from peripheral blood and tumor, suggests a strong association between the *RARB2* methylated promoter (Fang, Jian, et al., 2015) and *GSTP1* methylated promoter and breast cancer (Fang, Wei, et al., 2015). The lack of association between methylation of *RARB* and breast cancer in Peruvian women does not support these claims. On the other hand, *GSTP1* methylation was associated with breast cancer. Interestingly, GSTP1 has been found unmethylated and overexpressed in lung and breast cancer (Gurioli et al., 2018). Our results may reflect this methylation heterogeneity in the CpG islands that have been documented for the GSTP1 promoter and needs further investigation (Grenaker Alnaes et al., 2015).

Nevertheless, when considering the PMR of *RARB* and *GSTP1* simultaneously to calculate the risk, significant OR values (*p* = 0.040) were obtained for the qualitative PMR. In our sample of Peruvian women, breast cancer patients were almost three times more likely to have been exposed to *RARB* or *GSTP1* methylation than controls. These findings support the use of methylation panels should be recommended for liquid biopsies assessment, where biomarker screenings are still in an early developmental phase (Loke & Lee, 2018). Using a panel is justified because the *GSTP1* methylated promoter shows a high specificity as a marker but a low sensitivity when evaluated in patients and controls. Therefore, its use combined with other markers such as *RARB2* is recommended (Gurioli et al., 2018). Nonetheless, false‐negative results studying biomarkers in liquid biopsies samples can be overcome using more sensitive techniques like digital PCR as seen in recent studies (Cui et al., 2018; Weisenberger et al., 2008).

Regarding the methylation of *RARB* and *GSTP1* in controls in the Fujita et al. study, *GSTP1* methylated promoter was found in two controls and *RARB* maintained the highest sensitivity, without amplifying in the control group (Fujita et al., 2012). However, it is common to find a low degree of methylation in controls for *RARB* and *GSTP1*, as seen in previous studies (Fang, Jian, et al., 2015; Grenaker Alnaes et al., 2015). This would be indicative of methylation levels in cells due to environmental factors (Fujita et al., 2012).

We found an association between the *RARB + GSTP1* PMR and healthy women younger than 50 years. This group of young women may have been exposed to hormones such as estradiol, common in hormonal contraceptives (Widschwendter et al., 2008). This possibility adds to the hormonal development factors of each participant (menarche age, age of first pregnancy) that were not considered in clinical history and could partially explain the presence of methylated *RARB* (Xu et al., 2013).

According to the evidence presented in the meta‐analysis of *RARB* (Fang, Jian, et al., 2015) and *GSTP1* (Gurioli et al., 2018), together with the results obtained in the present study, it is possible that methylated women in the control group may have a higher risk of developing breast cancer after menopause.

In the patient group, no association was found between PMR values of *RARB* and *GSTP1* and clinicopathological variables. This finding is in accordance with Fang et al. meta‐analysis (Fang, Jian, et al., 2015; Fang, Wei, et al., 2015). Our findings support the evidence that *RARB* and *GSTP1* methylation, being an initial modification in carcinogenesis, occurs very early in breast cancer and not during its development (Fang, Jian, et al., 2015). Consequently, no association of these methylated genes with cancer stages was found. There were also no differences between the presence of methylation of these genes and immunohistochemical variables such as ER, PR, and HER2 or the molecular subtypes, in agreement with previous studies (Fujita et al., 2012, 2014).

Also, a significant association of *RARB + GSTP1* and patients over age 50, along with postmenopausal status was observed. This finding reinforces the hypothesis that age‐related changes in methylation and gene expression could explain, in part, the fact that advanced age and hormonal exposure are risk factors for developing breast cancer (Song et al., 2017). Considering the low frequency of methylated samples in our study, the sensitivity of *RARB* (20.69%), GSTP1 (10.34%), and *RARB + GSTP1* (31.03%) shows the difficulties of the *Methylight* assay to correctly classify breast cancer patients parting from free circulating bisulfite‐converted DNA, a situation that could be improved using higher initial plasma volume (>1.5 mL) or a more sensitive technique like digital PCR (Cui et al., 2018).

Regarding the specificity of the *Methylight* assay, it was the PMR of *GSTP1* that obtained the highest specificity (98.28%). The specificity of the methylated promoter of *RARB* (87.93%) briefly decreases when both genes are combined (86.21%). These results indicate that the methylation panel may confirm sick individuals. However, since methylation was found in controls, it would not be exclusive to the tumor cell, and this impairs the specificity of the test. Considering the predictive values of the panel, obtaining a negative result in the *Methylight* trial would not allow to safely rule out disease‐associated methylation (NPV = 55.56), while a positive result will not suffice to confirm the diagnosis, but may be used as an indication for additional tests (PPV = 69.23). The latter is valid for *RARB* and *GSTP1*, the results of sensitivity and specificity obtained being comparable with the revisions consulted (Fang, Jian, et al., 2015; Fang, Wei, et al., 2015; Gurioli et al., 2018).

The use of Methylight assay in a real clinical scenario requires appropriate infrastructure and trained personnel. In Peru, the need for specialized and certified molecular biology laboratories might be partially solved by the creation of Directive N°054‐INS/CNSP‐V.02, which aimed to strengthen the molecular detection of SARS‐CoV‐2 virus by monitoring and certifying public and private laboratories (Instituto Nacional de Salud, n.d.). It is still unknown if these efforts might be sustained in future times to allow the infrastructure to be used for molecular diagnosis of other diseases, including the implementation of biomarker tests for breast cancer.

In conclusion, our study accounts for the first evidence of the association of promoter hypermethylation of *RARB2* and *GSTP1* genes in liquid biopsies (circulating cfDNA) and breast cancer in Peruvian women. These biomarkers of liquid biopsies have the potential to be used in a comprehensive panel along with other genetic and epigenetic markers. We suggest the use of more sensitive techniques to discriminate the presence of methylated markers as liquid biopsy is a promising tool for early diagnosis. Finally, future studies with larger populations should be performed to explore the breast tumor methylome of patients aiming to identify and validate promising epigenetic biomarkers that may improve diagnostic panels and use them as a tool for early detection of breast cancer.

## AUTHOR CONTRIBUTIONS

Conception and design: JB, OAC, PD. Sample recollecting and selection: JMC, HGM, SGL. Data analysis and interpretation: JB, OAC, AGMC, PD, JP, JA. Statistical analyses: OAC, PD. Manuscript writing: PD, MLG, JB, RF. Final approval of manuscript: OAC, AGMC, JMC, SGL, HGM, RF, MLG, JB, PD.

## FUNDING INFORMATION

This study was funded by research grants from the Facultad de Medicina Humana de la Universidad de San Martín de Porres (Project E10012016038), Programa Nacional de Innovación para la Competitividad y Productividad—Innóvate Perú (Grant Number 138‐PNICP‐PIAP‐2015), Oncosalud‐AUNA, and Origenetica SAC.

## CONFLICT OF INTEREST STATEMENT

The authors declare no potential conflicts of interest.

## Supporting information


**Table S1.** Sample size calculation for matched case‐control study.

**Table S2.** Identifiers, primers, and probes used to amplify the methylated promoter of RARB and GSTP1 in the Methylight assay.

**Table S3.** Amplification conditions for the Methylight assay.

**Table S4.** Differences between breast cancer patients and healthy controls for the technical study variables.

## Data Availability

Data is available in the article's supplementary material.
