# Supplementary material for: Promoter hypermethylation of RARB and GSTP1 genes in plasma cell‐free DNA as breast cancer biomarkers in Peruvian women
Source: Mol Genet Genomic Med. 2023 Aug 7;11(12):e2260. doi: 10.1002/mgg3.2260 (PMC10724513; doi:10.1002/mgg3.2260)
Supplement: Supplementary file 1 — Table S1. Sample size calculation for matched case‐control study. [file MGG3-11-e2260-s003.docx]

| **Table S1. Sample size calculation for matched case control study** | | |
| --- | --- | --- |
| **Study Parameters** | ***RARB*** | ***GSTP1*** |
| Alpha | 0.05 | 0.05 |
| Power | 80% | 80% |
| Probability of exposure of cases | 37.32% | 33.45% |
| Expected Odds Ratio | 7.27 | 7.85 |
| **Required number of pairs 1:1** | **33** | **35** |
| **Sample size and power in study** | |  |
| Matched sample size 1:1 | **58** | **58** |
| Power | **97%** | **96%** |
