# Supplementary material for: Promoter hypermethylation of RARB and GSTP1 genes in plasma cell‐free DNA as breast cancer biomarkers in Peruvian women
Source: Mol Genet Genomic Med. 2023 Aug 7;11(12):e2260. doi: 10.1002/mgg3.2260 (PMC10724513; doi:10.1002/mgg3.2260)
Supplement: Supplementary file 2 — Table S2. Identifiers, primers, and probes used to amplify the methylated promoter of RARB and GSTP1 in the Methylight assay. [file MGG3-11-e2260-s002.docx]

| **Table S2. Identifiers, primers and probes used to amplify the methylated promoter of *RARB and GSTP1* genes in the Methylight assay** | | | | | | | | | | | | | |
| --- | --- | --- | --- | --- | --- | --- | --- | --- | --- | --- | --- | --- | --- |
| **HGNC gene symbol** | **Name** | **OMIM accession number** | **Gene ID** | **RefSeq Gene** | **GenBank version number*** | **Oligonucleotides** |  | **SEQUENCE 5’->3’** |  | **AMPLICON SEQUENCE** | **SIZE** | **UBICATION OF STUDIED REGION** | **EXTENSION OF CpG ISLAND** |
| *COL2A1* | Collagen Type II Alpha 1 | 120140 | 1280 | NG_008072.1 | NC_000012.12 | **PROBE** |  | CCTTCATTCTAACCCAATACCTATCCCACCTCTAAA* |  | GGGAAGATGGGATAGAAGGGAATATATTTAGAGGTGGGGATAGGTATTGGGTTAGAATGAAGGTTTGGTGGTTGGAGTTTATAATTGTTAGA | **92** | **GRCh38.p14 Chromosome12** | ***** |
|  |  |  |  |  |  | **FORWARD** |  | GGGAAGATGGGATAGAAGGGAATAT* |  |  |  |  |  |
|  |  |  |  |  |  | **REVERSE** |  | TCTAACAATTATAAACTCCAACCACCAA* |  |  |  |  |  |
| *GSTP1* | Glutathione S-transferase pi 1 | 134660 | 2950 | NG_012075.1 | NC_000011.10 | **PROBE** |  | ATAAGGTTCGGAGGTCGCGAGGTTTTCGT* |  | CGTCGTGATTTAGTATTGGGGCGGAGCGGGGCGGGATTATTTTTATAAGGTTCGGAGGTCGCGAGGTTTTCGTTGGAGTTTCGTCGTCGTAGTTTTCGTTATTAG | **105** | **GRCh38.p14 Chromosome11** | **[67350833 - 67351740]** |
|  |  |  |  |  |  | **FORWARD** |  | CGTCGTGATTTAGTATTGGGGC* |  |  |  |  |  |
|  |  |  |  |  |  | **REVERSE** |  | CTAATAACGAAAACTACGACGACGAAA* |  |  |  |  |  |
| *RARB* | Retinoic Acid Receptor Beta | 180220 | 5915 | NG_029013.3 | NC_000003.12 | **PROBE** |  | AGGCGTAAAGGGAGAGAAGTTGGTGTTTA* |  | GAATATCGTTTTTTAAGTTAAGTCGTCGTAAATAAAAAGGCGTAAAGGGAGAGAAGTTGGTGTTTAACGTGAGTTAGGAGTAGCGTTTC | **89** | **GRCh38.p14 Chromosome 3** | **[25465000 - 25480000]** |
|  |  |  |  |  |  | **FORWARD** |  | GAATATCGTTTTTTAAGTTAAGTCGTC* |  |  |  |  |  |
|  |  |  |  |  |  | **REVERSE** |  | GAAACGCTACTCCTAACTCACG* |  |  |  |  |  |
| *Oligonucleotides designed from sequences available in Fujita et al. 2014. | | | | | | | | | | | | | |
