# Supplementary material for: Promoter hypermethylation of RARB and GSTP1 genes in plasma cell‐free DNA as breast cancer biomarkers in Peruvian women
Source: Mol Genet Genomic Med. 2023 Aug 7;11(12):e2260. doi: 10.1002/mgg3.2260 (PMC10724513; doi:10.1002/mgg3.2260)
Supplement: Supplementary file 3 — Table S3. Amplification conditions for the Methylight assay. [file MGG3-11-e2260-s004.docx]

| **Table S3. Amplification conditions for the *Methylight* assay** | | |
| --- | --- | --- |
| **Reactive** | **Concentration** | **Volume (µL)** |
| Nuclease free water | - | 4 |
| *Epitect Mehtylight* Qiagen | 2X | 10 |
| Primer Probe Mix | 10X | 2 |
| Bisulfite converted DNA | - | 4 |
| **Stage** | **Temperature** | **Time** |
| Hold | 95°C | 5 minutes |
| Denaturation | 95°C | 15 seconds |
| Annealing | 60°C | 30 seconds |
| Extension | 72°C | 30 seconds |
| Cycling number | 50 cycles | |
| Acquisition | During extension using FAM channel | |
