# Supplementary material for: Promoter hypermethylation of RARB and GSTP1 genes in plasma cell‐free DNA as breast cancer biomarkers in Peruvian women
Source: Mol Genet Genomic Med. 2023 Aug 7;11(12):e2260. doi: 10.1002/mgg3.2260 (PMC10724513; doi:10.1002/mgg3.2260)
Supplement: Supplementary file 4 — Table S4. Differences between breast cancer patients and healthy controls for the technical study variables. [file MGG3-11-e2260-s001.docx]

| **Table S4. Differences between breast cancer patients and healthy controls for the technical study variables** | | | | | |  |
| --- | --- | --- | --- | --- | --- | --- |
|  |  |  |  |  |  |  |
|  |  | **Controls n (%)** | **Patients n (%)** | **Test** | **p-value** |  |
| **N =116** | | **58 (50)** | **58 (50)** |  |  |  |
| **Initial DNA mass (ng)** | | **n= 39** | **n= 36** |  | |  |
|  | Mean | 427.87 | 455.45 | 0.001 | 1.00** |  |
|  | Median | 399.90 | 424.00 |  |  |  |
|  | 95% CI | [ 399.5-402.9] | [298.98-515.72] |  |  |  |
|  | Range | [156.25-1292] | [99.84-1268] |  |  |  |
| ***COL2A*1 (ng/reax)** | | **n= 58** | **n= 58** |  | |  |
|  | Mean | 0.45 | 0.47 | 0.069 | 0.945** |  |
|  | Median | 0.33 | 0.39 |  |  |  |
|  | 95% CI | [0.2 - 0.4] | [0.24 - 0.5] |  |  |  |
|  | Range | [0.01 - 1.65] | [0.01 - 4.46] |  |  |  |
| **Age** | | **n= 58** | **n= 58** |  | |  |
|  | Mean | 52.93 | 52.93 | 0.0001 | 1.00* |  |
|  | Median | 52.00 | 52.00 |  |  |  |
|  | 95% CI | [49 - 56.9] | [49 - 57] |  |  |  |
|  | Range | [20 - 85] | [21 - 80] |  |  |  |
| **p-value obtained using Two-sample Wilcoxon rank-sum (Mann-Whitney) test. * According to Student's T test | | | | | |  |
